# Molecular determinants of TNFR1:TNFα binding and dynamics in a physiological membrane environment

**DOI:** 10.1016/j.crstbi.2025.100177

**Published:** 2025-12-18

**Authors:** Elena Álvarez Sánchez, Simon Huet, Stéphane Téletchéa

**Affiliations:** a Nantes Université, CNRS, UMR 6286, 2 rue de la Houssinière, Nantes, F-44000, France; b Affilogic SAS, 24 rue de la rainière, Nantes, F-44000, France

**Keywords:** TNF-α, TNF receptor 1, Molecular modelling, Membrane environment, GaMD

## Abstract

Tumor Necrosis Factor alpha (TNFα) is a pro-inflammatory cytokine critical for regulating cell survival and death. Under pathological conditions, excessive TNFα activity can lead to chronic inflammation, contributing to diseases such as inflammatory bowel disease and other autoimmune disorders. While structural studies have elucidated the atomistic details of TNFα binding to its receptor, TNF Receptor 1 (TNFR1), the influence of the membrane environment on this interaction remains poorly characterized experimentally. In this study, we employed advanced all-atom Gaussian accelerated molecular dynamics simulations to investigate how lipid-mediated interactions modulate the TNFα–TNFR1 complex. We identified key residues on both the cytokine and its receptor that govern trimer assembly, receptor binding, and potential pathological alterations. Our analysis confirmed previously identified functional sites and revealed new residues likely to contribute to the structural stability and dynamics of the complex. These findings provide a more comprehensive understanding of the molecular determinants of TNF signaling and offer a foundation for future experimental investigations into the receptor-ligand interface and membrane-mediated regulation.

## Highlights

• Molecular dynamics simulation of TNF-ɑ bound to a membrane-anchored TNFR1.
• Detailed MM/PBSA analysis allowed the identification of most contributing amino acids.
• Membrane anchoring impacts global motions of the system.
• Protein:lipids interactions extend beyond transmembrane region.
• Some of the most contributing amino acids are already linked to pathogenic mutations.

## Graphical abstract

**Figure:**
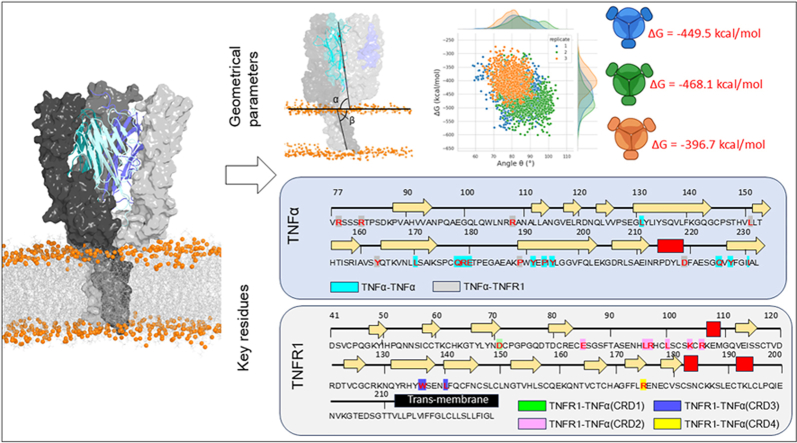

## 1 Introduction

Inflammation constitutes one of the first protective barriers of human immunity. The inflammatory response is characterized by the release of cytokines, such as Tumor Necrosis Factor alpha (TNFα). This pleiotropic cytokine was first discovered for its anti-tumoral effects in mice (Carswell et al., 1975). However further studies revealed that systemic administration of TNFα induces severe endotoxic effects (van Loo and Bertrand, 2023; Lejeune et al., 2006).

Excess amounts of TNFα expressed in cells have been mainly associated with the development of immune diseases (Palladino et al., 2003), in particular in psoriasis, Crohn's disease and rheumatoid arthritis. Consequently, targeting this unregulated TNFα has become a major focus of therapeutic research in order to counteract the inflammatory response involved in these diseases.

TNFα exists in two forms: the membrane-bound form (mbTNFα) and the soluble form (sTNFα). sTNFα is generated by proteolytic cleavage of the extracellular domain of mbTNFα by TNFα-converting enzyme (TACE), also known as disintegrin and metalloprotease domain 17 (ADAM17). TNFα is biologically active in its homotrimeric form (Smith and Baglioni, 1987).

This cytokine is produced by activated macrophages and other immune cells, and exerts its function by interacting with two receptors: TNFR1 and TNFR2. TNFR1 (p55), which is ubiquitously expressed and contains a death domain (DD), is primarily activated by sTNFα. Conversely, mbTNFα predominantly activates TNFR2 (p75), which is restricted to immune, neuronal, and endothelial cells. Importantly, the biological outcome of TNFα signaling is determined by both the form of the ligand and the receptor engaged (Ruder et al., 2019). TNFR2, which lacks a death domain, mediates in homeostatic, cell survival, migration and apoptotic pathways, as well as cell activation. In contrast, TNFR1 signaling is essential to induce cytotoxic and pro-inflammatory responses.

Given the central role of this cytokine and its receptors in regulating inflammation, dysregulation of this signaling axis has been implicated in several pathological conditions (Yang et al., 2018). One such example is TNF Receptor-1 Associated Periodic Syndrome (TRAPS) (McDermott et al., 1999), an autoinflammatory disorder caused by mutations in the TNFR1 gene. Although the underlying mechanisms remain incompletely understood, these variants may impair receptor folding and disrupt TNFα binding. Clinically, TRAPS is characterized by recurrent episodes of fever accompanied by systemic inflammatory manifestations. TNFα overexpression has also been directly implicated in chronic inflammatory diseases, including inflammatory bowel disease (IBD)—which encompasses Crohn's disease (CD) and ulcerative colitis (UC) (Aebisher et al., 2024)—, rheumatoid arthritis (RA) (Ding et al., 2023), and psoriasis (Jang et al., 2021). In these conditions, elevated levels of TNFα contribute to sustained inflammation and tissue damage. As such, therapeutic strategies targeting the TNFα:TNFR1 interaction have been developed to mitigate pathological inflammation. These include anti-TNFα monoclonal antibodies such as adalimumab (Hu et al., 2013), infliximab (Liang et al., 2013), and golimumab (Ono et al., 2018), as well as soluble TNF receptor fusion proteins like etanercept (Sandborn et al., 2001), which function as decoys to neutralize TNFα.

From a structural standpoint, each TNFα protomer adopts a "jelly-roll" type β-sandwich fold (Richardson, 1981), composed predominantly of anti-parallel beta sheets (Eck and Sprang, 1989). This topology features two five-stranded β-sheets, with the inner sheet forming the interface between the three protomers, also known as the TNFα homology domain (THD).

TNFR1 comprises an extracellular N-terminal domain and a cytoplasmic signaling domain, connected by a transmembrane helical segment. It belongs to both the "nerve growth factor receptor" superfamily (Naismith et al., 1995) and the TNF Receptor Superfamily (TNFRSF). Structurally, TNFR1 is elongated and consists of four cysteine-rich domains (CRDs), as illustrated in Fig. 1, each containing six conserved cysteines arranged in a configuration reminiscent of rungs of a twisted ladder (Banner et al., 1993).

**Fig. 1:**
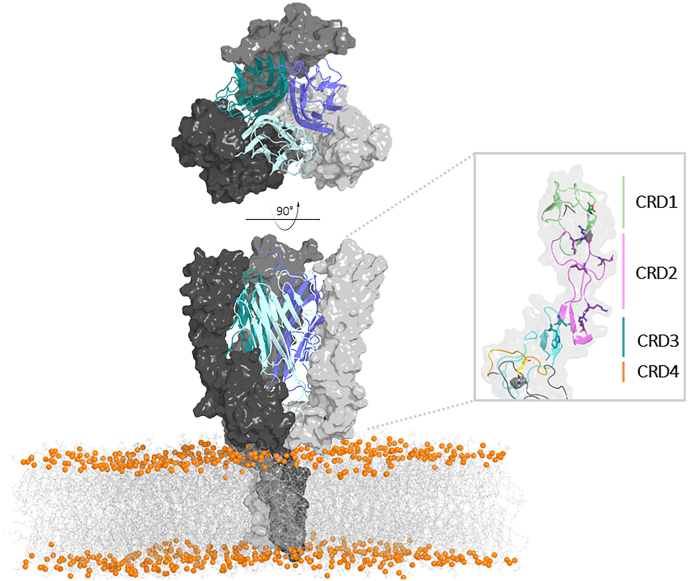
Structural organization of the trimeric TNFR1 receptor in complex with trimeric TNFα. The TNFR1 trimer is shown in surface representation (gray scale), bound to the TNFα homotrimer. The right panel displays the extracellular region of a single TNFR1 protomer, color-coded by cysteine-rich domain (CRD): CRD1 in green, CRD2 in magenta, CRD3 in blue, and CRD4 in orange.

In 1995, the crystal structure of TNFR1 revealed a dimeric interaction mediated by CRD1, known as the pre-ligand binding assembly domain (PLAD) (Naismith et al., 1995). This discovery prompted the hypothesis that TNFR1 preassembles as dimers on the cell surface. Upon engagement by trimeric TNFα, receptor trimerization and high-order oligomerization are believed to occur (Lewis et al., 2012). PLAD-mediated interactions may further stabilize receptor clustering, forming a geometrically organized network of complexes. This oligomerization is thought to be crucial for effective signal transduction, with CRD1 and CRD2 playing primary roles in ligand interaction. The resolution of a trimeric TNFα complex coexisting with TNFR1 dimers (7KP7) (McMillan et al., 2021) confirmed the persistence of TNFR1 dimers upon ligand binding. This structural insight corroborates the model in which TNFR1 activation occurs through the association of TNFR1:TNFα complexes into hexagonal networks, which are crucial for signal transduction via the recruitment of molecules such as TRADD, TRAF2 and RIPK1 (Lo, 2025).

In this study, motivated by the critical role of TNFα:TNFR1 interactions in inflammatory pathologies, we investigated the structural and dynamic features of this cytokine-receptor complex in a membrane environment using Gaussian accelerated Molecular Dynamics (GaMD). This approach allowed us to, at atomic resolution, study the conformational landscape of the trimeric ligand engaging its trimeric receptor, and to assess how membrane embedding influences the stability and geometry of the interaction. Moreover, we investigated in detail the intra-trimeric interface of soluble TNFα to better understand the determinants of its structural assembly. Together, these advanced simulations provide a detailed mapping of key interaction hotspots within the TNFα:TNFα and TNFR1:TNFα interfaces, offering mechanistic insights that may inform future therapeutic strategies targeting this signaling axis.

## 2 Material and methods

### 2.1 Modelling TNFR1:TNFα complex

Crystal structure 1EXT from TNFR1 extracellular region and 1TNF (Eck and Sprang, 1989) from TNFα were used to model the TNFR1:TNFα complex. The different structures were superimposed using TNFR1-TNFb complex (1TNR) and relaxed using the Rosetta Relax protocol (Kaufmann et al., 2010).

Transmembrane helices were modelled as an ideal helices using ROSETTA ReModel protocol (Huang et al., 2011). The resulting helices were superimposed on the NMR structure of TNFR1 transmembrane region (7K7A) to obtain the transmembrane region of the model. The linker connecting this region to the globular structure was generated using MODELLER ModLoop webserver (Fiser and Sali, 2003).

The PPM webserver was used to preorient the membrane (Lomize et al., 2012). PACKMOL-Memgen allowed the insertion of the system into the lipid bilayer made up of POPC molecules (Schott-Verdugo and Gohlke, 2019). The parametrization was performed with Lipid21 forcefield for the lipids (Dickson et al., 2022) and ff14sb forcefield for the proteins (Maier et al., 2015). The minimum distance to box border was set to 25 Å while the minimum distance between protein/membrane and upper box border was set to 20 Å. The system was neutralized with Na+ and Cl-ions and solvated in a cubic box with the TIP3P water model (Mark and Nilsson, 2002).

### 2.2 cMD simulations

Conventional Molecular Dynamics (cMD) simulations were performed using the AMBER 22 collection (Case et al., 2005, 2023). Initial coordinates were taken from human TNFα structure (1TNF). The simulations were performed with a 2fs time step, periodic conditions and 8 Å cut-off for long-range interaction computation, after PME was used with default parameters (Sagui et al., 2004). After minimization, the system was heated up to 100 K in 50 ps with a constraint of 20 kcal mol^−1^Å^−2^ on solute coordinates. A second heat was performed to reach 303.0 K with a constraint of 5 kcal mol^−1^Å^−2^ on solute coordinates. A density step in NVT conditions was run for 100 ps with restraints on the macromolecules coordinates of 5 kcal mol^−1^Å^−2^.

The unconstrained cMD production simulation was computed for 1000 ns for the system.

### 2.3 GaMD simulations

Gaussian accelerated Molecular Dynamics (GaMD) simulations (Miao and McCammon, 2017) were performed using the AMBER 22 collection (Case et al., 2005, 2023). Initial coordinates were taken from the model described above. The simulations were performed with a 2fs time step, periodic conditions and 8 Å cutoff for long-range interaction computation; after this distance PME was used with default parameters. After minimization, the system was heated up to 100 K in 50 ps with a constraint of 20 kcal mol^−1^Å^−2^ on solute coordinates. A second heat was performed to reach 303.0 K with a constraint of 5 kcal mol^−1^Å^−2^ on solute coordinates. A density step in NVT conditions was run for 100 ps with restraints on the macromolecules coordinates of 5 kcal mol^−1^Å^−2^. A 500 ns cMD equilibration was performed to further relax the system.

Subsequently, a 2 ns and 10 ns cMD unconstrained simulations were carried out to further relaxation and parameter collection. The ending structure was used for a 2 ns GaMD pre-equilibration followed by 40 ns GaMD equilibration applying force parameters. The unconstrained GaMD production simulation was then computed for 1000 ns in semi-isotropic conditions for each replicate.

### 2.4 Free energy calculations

For soluble TNFα and membrane systems, MM/PBSA binding energy calculations were performed on a single trajectory with MMPBSA.py (Miller et al., 2012) using mbondii2 atom types and igb 5 parameters. The entire simulation time was considered for energy calculation for sTNFα, with data sampled at one frame per nanosecond, excluding ions and solvent molecules, which corresponds to 1000 frames, "memopt" parameter was set to 1.

Per-residue energy decomposition was performed to determine individual contributions at interface between TNFR1 and TNFα or between TNFα protomers.

### 2.5 Trajectory analysis

CPPTRAJ was used for removing system periodicity, rotation, translation and for trajectory analysis (Roe and Cheatham, 2013). The resulting trajectory contained one frame per nanosecond. Principal Component Analysis was performed using the Bio3D R package (Grant et al., 2021), with lipids excluded from the analysis. The PyLipID Python package (Song et al., 2022) was used to study protein-lipid interactions during the last 100 ns of the simulation. For analyzing the most conserved interactions between TNFR1 and TNFα, Residue Interaction Networks were analyzed using RING 4.0 (Del Conte et al., 2024). The HELANAL method (Bansal et al., 2000), from MDAnalysis (Gowers et al., 2016; Michaud-Agrawal et al., 2011) was used for transmembrane helices parameters monitoring.

## 3 Results

### 3.1 Energetic analysis of TNFα, a trimeric ligand

Before studying the interaction between trimeric TNFR1 and trimeric sTNFα in a membrane context, we first sought to characterize the energetic features of the interaction between the protomers constituting the cytokine. This allowed us to identify the key residues contributing to protomer–protomer interactions within the TNFα trimer. Using the MM/PBSA approach, we applied a threshold of −2 kcal/mol to define residues with significant energetic contributions to the interface. Based on this criterion, twelve residues were identified (Table 1), among which three (R179, Y191, and Y195) exhibited particularly strong contributions, with respective interaction energies of −9.3, −4.4, and −4.6 kcal/mol.

**Table 1:** Summary of the 12 residues that showed a contribution to the interaction of a TNFα protomer with the other two protomers, with values less than −2 kcal/mol. The total contribution for each of these residues is displayed, along with the electrostatic, polar,and van der Waals components, sorted by the average total ΔG contribution.

TNFα residue	Avg total contribution	Avg VDW contribution	Avg polar contribution	Avg electrostatic contribution
R82	−3.47 ± 4.44	−1.49 ± 1.38	50.04 ± 30.99	−52.02 ± 33.36
L131	−3.14 ± 0.76	−3.67 ± 0.64	1.09 ± 0.65	−0.56 ± 0.72
L170	−2.70 ± 0.57	−2.78 ± 0.43	1.84 ± 0.27	−1.76 ± 0.39
Q178	−2.32 ± 2.76	−4.70 ± 0.95	12.69 ± 2.59	−10.31 ± 4.59
R179	−9.30 ± 2.88	−3.80 ± 1.31	43.32 ± 10.30	−48.80 ± 12.69
E180	−2.8 ± 4.30	−0.18 ± 1.31	42.97 ± 9.45	−45.6 ± 11.44
Y191	−4.43 ± 0.78	−5.46 ± 0.61	6.44 ± 0.64	−5.42 ± 0.89
P193	−2.60 ± 0.41	−2.87 ± 0.42	0.86 ± 0.65	−0.58 ± 0.60
Y195	−4.55 ± 1.04	−6.06 ± 0.57	4.02 ± 0.56	−2.52 ± 1.11
Q225	−2.20 ± 0.71	−2.73 ± 0.41	2.32 ± 0.95	−1.79 ± 1.34
Y227	−2.50 ± 0.84	−2.98 ± 0.46	1.97 ± 0.84	−1.48 ± 1.04
I231	−2.05 ± 0.39	−2.19 ± 0.36	0.72 ± 0.24	−0.57 ± 0.24

Since this interface constitutes the TNFα Homology Domain (THD), we observed a combination of hydrophobic and polar residues contributing to its stability (Fig. 2A). Among them, three tyrosines (Y191, Y195, Y227), two leucines (L131, L170), and one isoleucine (I231) are located on the four-stranded β-sheet that forms the core of the interface. In complement, charged and polar residues, including R82, R179, E180, Q179, and Q225, are mainly located at the periphery of this β-sheet, where they likely contribute to the cytokine solubility. We found R82, R179, E180, Q179, and Q225. Additionally, the proline P193 satisfies the energy threshold and completes the list of the twelve top contributing residues.

**Fig. 2:**
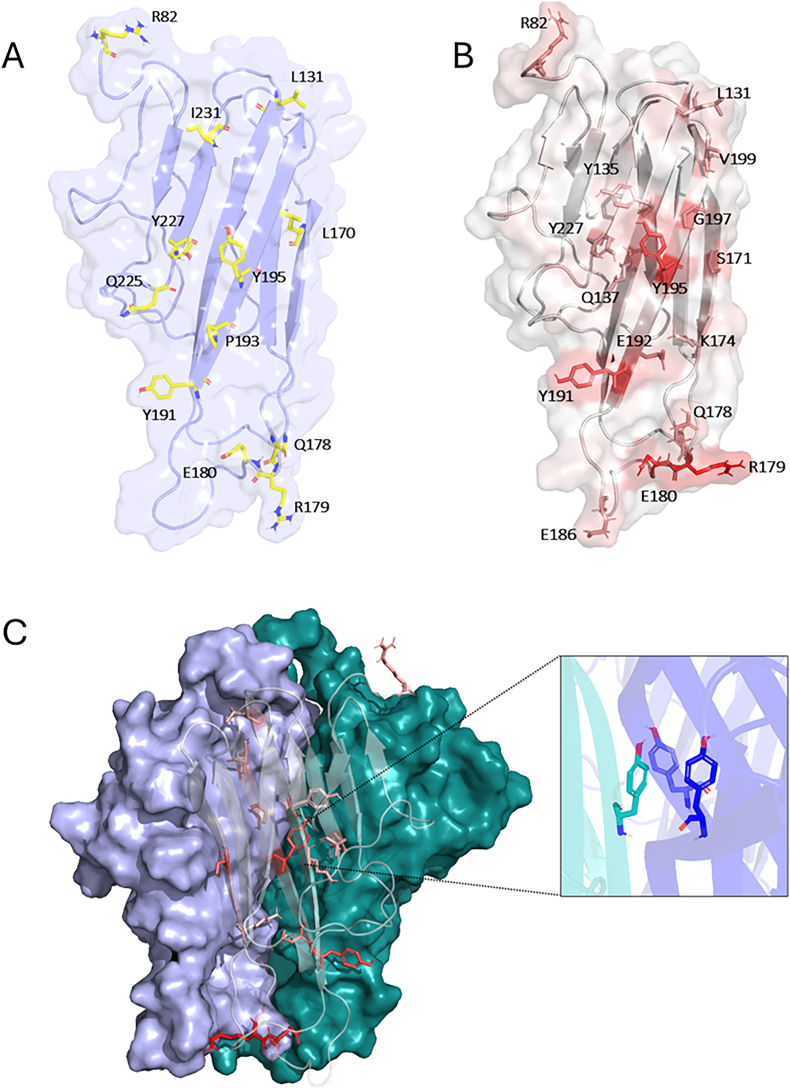
Structural analysis of TNFα protomer interfaces. (A) Cartoon representation of a single TNFα protomer with transparent surface rendering. The twelve key interfacial residues identified via the MM/PBSA analysis are shown as yellow sticks. (B) Same representation as in (A), highlighting residues involved in conserved inter-protomer interactions identified using RING 4.0, shown as colored sticks on a white-to-red scale indicating conservation frequency. (C) Surface representation of the TNFα trimer, with one protomer shown in cartoon. A zoomed-in view highlights the conserved interaction between the three Y191 residues across the protomers.

To analyze the most conserved interactions involving these residues, we employed the RING 4.0 tool. Fig. 2B highlights the residues involved in highly conserved inter-protomer interactions along the trajectory. Several of the previously identified key contributors, namely Y195, Y191, E180, and R179, were found to participate in highly conserved contacts between protomers. In addition, RING analysis revealed other residues not included among the top 12 energetic contributors, including Y135, Q137, V199, G197, S171, K174 and E186. These residues showed weaker contributions, with interaction energies above the −2 kcal/mol threshold.

Among all identified residues, Y191 and Y195 emerge as particularly important for trimer stabilization, contributing −4.6 and −4.4 kcal/mol, respectively. Both also showed substantial van Der Waals contributions, at −6.1 and −5.5 kcal/mol. Furthermore, RING 4.0 analysis revealed a conserved network of П stacking interactions specifically involving the Y195 residues across the three protomers (Fig. 2C). The residue Q178, located within a loop connecting β-strands, contributed −2.3 kcal/mol in total, with a Van Der Waals component of −4.7 kcal/mol. The residue R179 displayed the strongest energetic contribution among all residues, for a total of −9.3 kcal/mol, with Van Der Waals, polar and electrostatics components of −3.80, 43.32 and −48.80 kcal/mol respectively. Moreover, R179 forms highly conserved inter-protomer interactions with E180, underscoring its central role in trimer stability.

### 3.2 Detailed analysis of the interface between TNFR1 and TNFα

As previously discussed, TNFα, a trimeric cytokine, engages TNFR1 by promoting its trimerization and subsequent oligomerization. To characterize the molecular determinants of this interaction, we performed MM/PBSA-based free energy calculations with per-residue decomposition.

The total binding free energy varied across replicates, with values of −449.5 ± 49.01 kcal/mol, −468.1 ± 50.6 kcal/mol, and −396.68 ± 39.99 kcal/mol for replicates 1, 2, and 3, respectively. These differences likely reflect the dynamic nature of the membrane environment and the variability in contacts formed between TNFα and TNFR1 across the simulation runs.

To investigate the contribution of individual residues, we focused on the extracellular portion of TNFR1, comprising the four cysteine-rich domains (CRDs). Residue-wise contributions were analyzed for each chain and replicate, as shown in Fig. S1. Using a threshold of −2 kcal/mol to define energetically significant contributors, we identified ten residues with average contributions exceeding this cutoff. These include one residue in CRD1 (D71), five in CRD2 (E85, L96, R97, L100, and K104), two in CRD3 (W136 and L140), and one in CRD4 (R175). We next examined how the contributions of these residues varied across chains and replicates. As illustrated in Fig. 3A, residues L96, L100, K104, W136, and L140 displayed highly conserved contributions across all chains and replicates. These residues are remarkably present in CRD2 and CRD3 (Fig. 3B), domains previously implicated in ligand binding. Detailed energetic values for these residues are provided in Table 2. To assess these residue contributions, we also performed a Residue Interaction Network analysis, shown in more detail in Fig. 4A–E and Table S2.

**Fig. 3:**
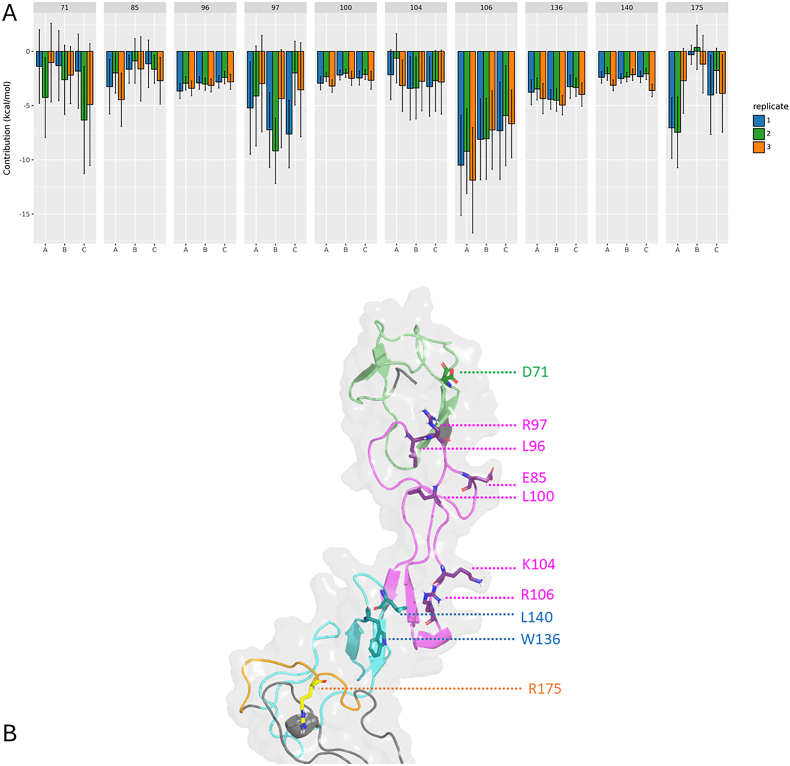
TNFR1 residues with a replicate averaged contribution to the interaction with TNFα per chain below −2 kcal/mol (A). Replicates 1, 2 and 3 are colored in blue, green and orange respectively. Residue 71 belongs to CRD1, residues 85, 96, 97, 100 and 104 to CRD2. Residue 106 and 136 are part of CRD3 while R140 belong to CRD4 (B).

**Table 2:** Detailed energetics contributions for total, electrostatics, polar, and van der Waals components for TNFR1. The average contributions were calculated for each residue for the different replicates. The corresponding values are sorted by the average ΔG value.

TNFR1 residue	Avg total contribution	Avg VDW contribution	Avg polar contribution	Avg electrostatic contribution	CRD
R106	−8.33 ± 4.08	−2.77 ± 1.16	55.49 ± 8.69	−61.06 ± 11.54	CRD2
R97	−5.15 ± 3.86	−3.52 ± 1.25	3.49 ± 11.15	−5.12 ± 13.53	CRD2
W136	−4.01 ± 1.04	−5.72 ± 0.90	5.84 ± 1.21	−4.13 ± 1.54	CRD3
R175	−3.12 ± 2.66	−1.4 ± 1.03	36.33 ± 9.56	−38.05 ± 10.9	CRD4
L96	−3.02 ± 0.62	−3.7 ± 0.49	1.88 ± 0.72	−1.19 ± 1.19	CRD2
D71	−2.88 ± 3.75	−0.6 ± 1.01	42.5 ± 14.18	−44.78 ± 15.9	CRD1
K104	−2.71 ± 2.66	−3.01 ± 1.05	50.18 ± 17.93	−49.88 ± 19.23	CRD2
L140	−2.52 ± 0.52	−2.66 ± 0.51	1.48 ± 0.53	−1.34 ± 0.54	CRD3
L100	−2.5 ± 0.56	−2.83 ± 0.50	1.94 ± 0.82	−1.61 ± 0.84	CRD3
E85	−2.16 ± 2.09	−1.34 ± 0.93	45.4 ± 16.53	−46.22 ± 17.52	CRD2

**Fig. 4:**
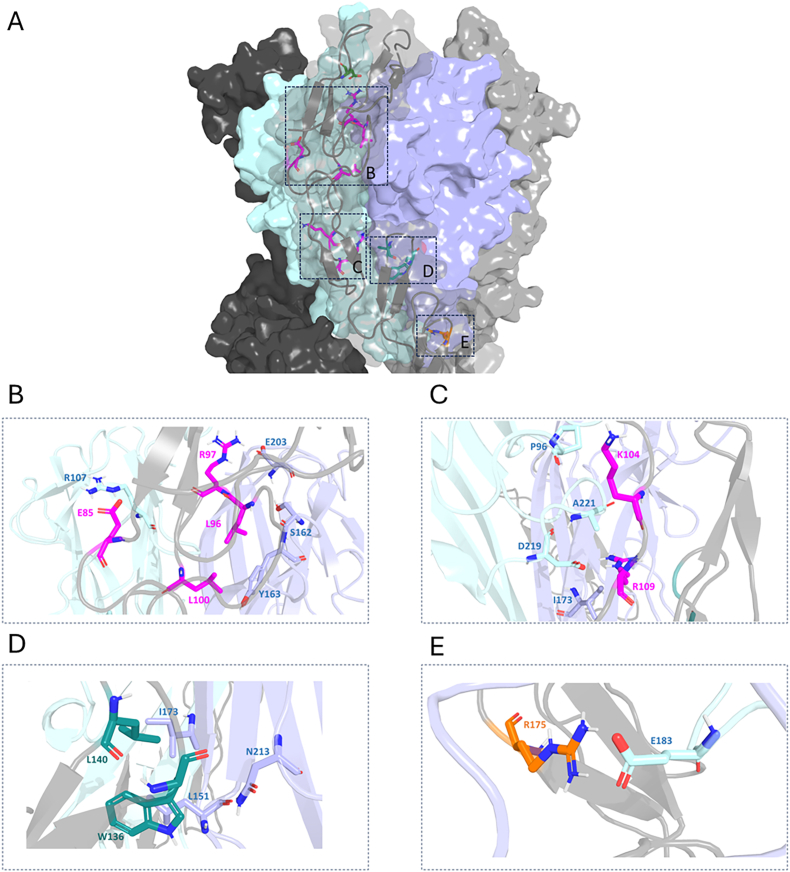
Global view of the contributing residues, colored by Cystein Rich Domain membership. Residues belonging to CRD1 are shown in green, magenta for CRD2, blue and orange for CRD3 And CRD4 respectively (A). Zoom views for conserved interactions between TNFR1 identified contributing residues and TNFα residues in regions (B), (C), (D) and (E).

D71, located in CRD1, displays an average contribution of −2.86 kcal/mol, with some variability across chains. Analysis of conserved interactions revealed that D71 forms hydrogen bonds with E183 of TNFα. Energetic decomposition indicates a strong electrostatic contribution (−61.06 kcal/mol), largely counterbalanced by a polar solvation component (55.49 kcal/mol), alongside a minor van der Waals (VDW) contribution of −0.6 kcal/mol.

CRD2 contains the largest number of contributing residues, with E85, L96, R97, L100, and K104 exhibiting average contributions of −2.16, −3.02, −5.15, −2.5, and −2.71 kcal/mol, respectively. E85 forms a hydrogen bond with R107, while L96 interacts with Y163 via van der Waals contacts (Fig. 4B and C). The average VDW contributions for these residues are −1.34, −3.70, −3.52, −2.83, and −3.01 kcal/mol, respectively.

Within CRD3, W136 and L140 are the main contributors. W136 shows an average contribution of −4.01 kcal/mol, with highly conserved involvement across chains and replicates. The VDW contributions for W136 and L140 are −5.72 and −2.66 kcal/mol, respectively, supporting their stable engagement at the interface (Fig. 4D).

Finally, R175 is the sole residue in CRD4 exceeding the energetic threshold. Although its contribution varies across chains and replicates, markedly negative values were observed, indicating a potential role in receptor–ligand stabilization (Fig. 4E).

**Fig. 5:**
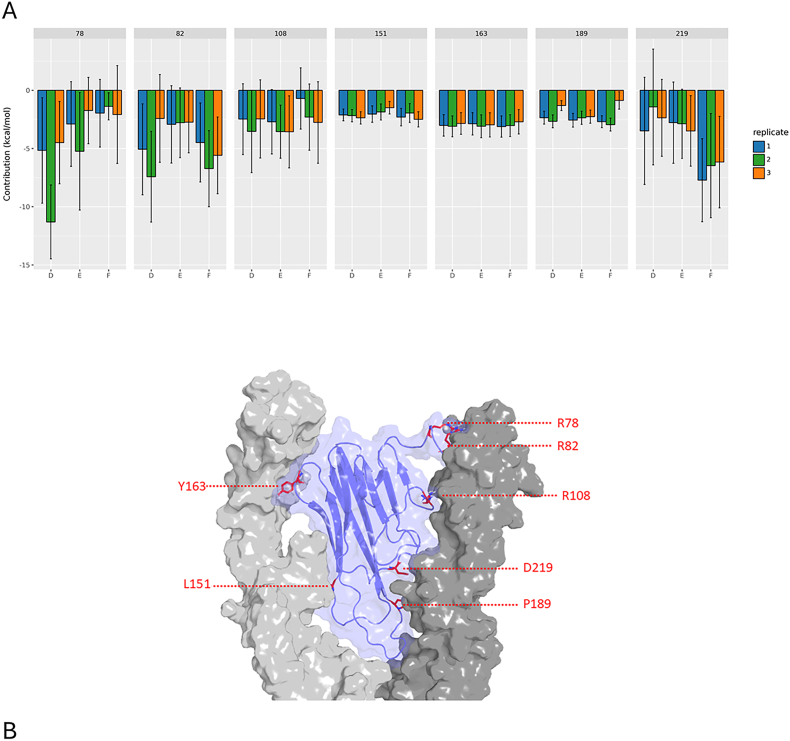
TNFα residues with a replicate averaged contribution to the interaction with TNFR1 per chain below −2 kcal/mol. Replicates 1, 2 and 3 are colored in blue, green and orange respectively. (A). Location of the most contributing residues (shown in red sticks). TNFα is shown in blue surfaces, TNFR1 in gray (B).

Regarding TNFα, 7 residues showed an average contribution below −2 kcal/mol (Fig. 5 and Table 3). In interaction with one TNFR1 protomer, we identified R78, R82, R108, D219, and P189 with respective contributions of −4.03 ± 3.45, −4.46 ± 3.39, −2.67 ± 3.01, −4.09 ± 3.82 and −2.23 ± 0.56 kcal/mol. This interface is notably enriched in charged residues. On the opposite face, which engages the other TNFR1 protomer, L151 and Y163 contribute −2.09 ± 0.63 and −2.97 ± 0.96, respectively. For these two residues, van der Waals interactions are predominant, with average contributions of −3.08 ± 0.57 kcal/mol for L151 and -6.07 ± 0.7 kcal/mol for Y163.

**Table 3:** Detailed energetics contributions for total, electrostatics, polar, and van der Waals components for TNFα. The average contributions were calculated for each residue for the different replicates. The corresponding values are sorted by the average ΔG value.

TNFα residue	Avg total contribution	Avg VDW contribution	Avg polar contribution	Avg electrostatic contribution
R82	−4.46 ± 3.39	−2.2 ± 1.37	32.98 ± 10.15	−35.25 ± 11.63
D219	−4.09 ± 3.82	0.02 ± 0.82	33.12 ± 6.53	−37.24 ± 7.49
R78	−4.03 ± 0.45	−2.47 ± 1.54	35.04 ± 15.14	−36.61 ± 17.0
Y163	−2.97 ± 0.96	−6.07 ± 0.7	5.25 ± 1.09	−2.15 ± 0.89
R108	−2.67 ± 3.01	−4.15 ± 1.23	40.38 ± 9.17	−38.9 ± 10.86
P189	−2.23 ± 0.56	−2.25 ± 0.49	0.26 ± 0.5	−0.25 ± 0.53
L151	−2.09 ± 0.63	−3.08 ± 0.57	0.47 ± 0.67	0.52 ± 0.56

### 3.3 Impact of membrane embedding on Receptor:ligand motion

**Fig. 6:**
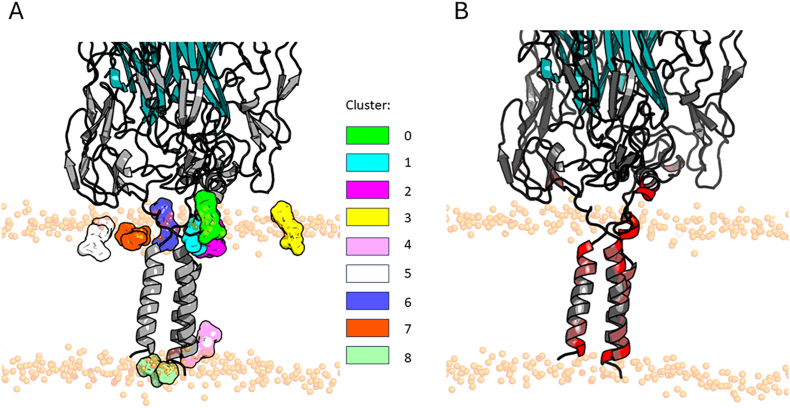
Protein-PC binding sites top ranked solutions per cluster provided by PyLipID analysis. Each PC molecule from the top ranked solution is shown in surface representation. Binding sites 0, 1, 2, 3, 4, 5, 6, 7 and 8 are colored in green, cyan, magenta, yellow, pink, gray, purple, orange and lightgreen respectively (A). Structure of TNFR1 in complex with TNFα colored by protein-PC interaction residence time (B). Lipids residence time corresponds to the duration of binding of PC to any amino acid. Amino acids with a long residence time with PC are colored in red.

Although the transmembrane helices are the most exposed to the lipid environment, lipid:protein interactions can extend beyond these regions. We analyzed the interactions between the protein and phosphatidylcholine (PC) lipid headgroups with PyLipID. As shown in Fig. 6A, eight PC:protein interaction clusters were identified, most of which are located near the linkers connecting the transmembrane and globular domains. This observation is reinforced by Fig. 6B, which highlights the long residence times of PC molecules at these linker regions. The amino acid composition of each cluster varies depending on its location and structural environment.

As detailed in Table 4, cluster 4 exhibited the largest lipid-binding surface area (22.87 nm^2^) and a residence time of 99 ns, involving 18 hydrophobic residues. This is consistent with its localization within the transmembrane region, which is characteristically enriched in hydrophobic amino acids.

**Table 4:** Lipid clusters information provided by PyLipID python package. The calculation was performed using the last 100 ns of the trajectory. Special residues refers to cystein, glycine and proline.

Cluster	0	1	2	3	4	5	6	7
**BS duration (ns)**	13.33	23.18	22.51	8.87	21.79	7.12	8.0	15.9
**BS occupancy (%)**	100	100	100	85	100	98	10	100
**BS residence time (ns)**	99	99	62.64	22.15	99	9.57	8.53	99
**Lipid count**	4.6	7.8	8.45	6.05	12.59	3.77	1.1	9.49
**BS surface area (nm^2^)**	9.52	13.90	16.68	16.47	22.86	10.30	1.61	18.37
**Charged residues**	2/13	3/20	8/19	4/25	0/23	1/14	1/5	4/29
**Polar residues**	3/13	5/20	4/19	7/25	2/23	5/14	0/5	10/29
**Hydrophobic residues**	4/13	7/20	6/19	6/25	18/23	4/14	2/5	10/29
**Special residues**	4/13	5/20	1/19	8/25	3/23	4/14	2/5	5/29

This work was partially sponsored by the Agence Nationale de la Recherche et de la Technologie (ANRT) via a Convention Industrielle de Formation par la Recherche (CIFRE n◦2022-104). Affilogic SAS, Nantes, France, provided financial support in the form of salaries for authors Elena Álvarez Sánchez and Simon Huet.

In contrast, cluster 2, with a binding surface area of 16.68 nm^2^ and a residence time of 62.64 ns, involves four distinct charged residues (E206, D207, K187, K186). These residues, located near the membrane, are well-positioned to interact with the polar headgroups of PC lipids. Detailed information on the residues contributing to each cluster is provided in Table S1. Cluster 7 involves the highest number of residues, 29 in total. This cluster, which has a binding surface area of 18.37 nm^2^, is comparable to cluster 2 in both location and composition but is situated on a different TNFR1 protomer. It also includes charged residues such as E206, D207, and K203. Cluster 3 comprises five charged residues (K193, K203, E190, K187, and E200). Notably, six cysteines (C149, C191, C168, C166, C185, and C182), most of them located beyond CRD4, also contribute to interactions with PC molecules.

**Fig. 7:**
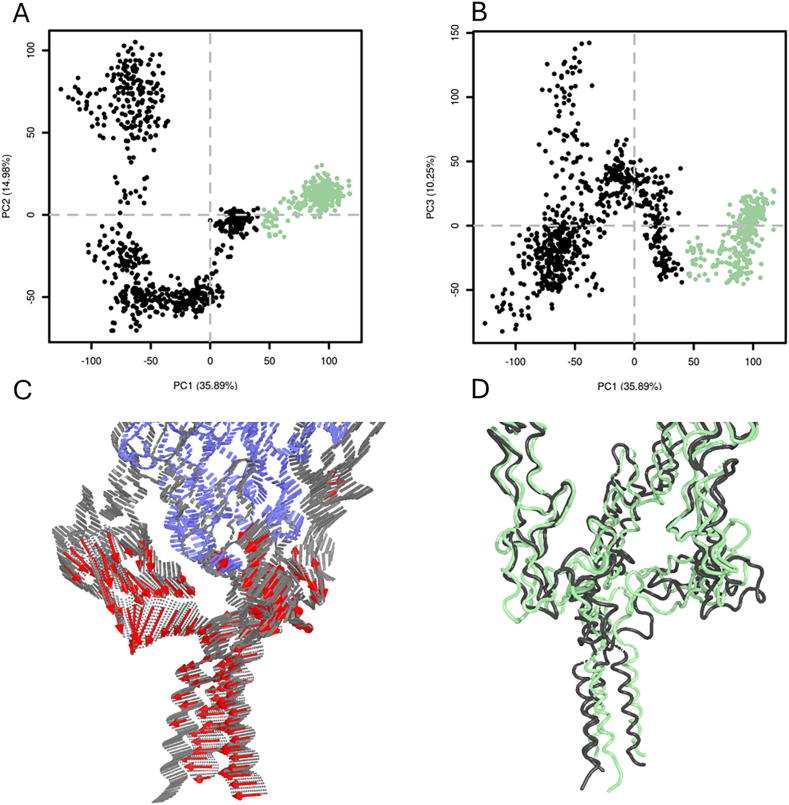
Results obtained from the PCA performed on 1000 ns the first replicate. Representation of the main motions explained by PC1 (A) and PC2 (B). Vectors representing the main motion of the system (red arrows) (C). Clusters centers for the two clusters identified after PCA analysis. TNFR1 structures are colored in black for cluster 1 and in green for cluster2 (D).

These clusters, positioned at the interface between the membrane and the globular region of TNFR1 and involving charged residues in the linker segments, appear to play a critical role in stabilizing the receptor's anchoring with the membrane. To further investigate how such interactions may affect the overall dynamics of the system, we performed a principal component analysis (PCA). The first three components accounted for 61.1 % of the total variance, and the projection of component 2 versus component 1 revealed two distinct conformational clusters (Fig. 7A and B). The dominant motions described by PCA (Fig. 7C) indicate pronounced flexibility of the linker regions toward the membrane, while the globular domains remained comparatively rigid. Analysis of representative structures from each cluster (Fig. 7D) confirmed that the most significant conformational variability stems from linker mobility. Additionally, a lateral displacement of the transmembrane helices along the membrane surface was observed.

Altogether, these results indicate that membrane-associated protein–lipid interactions, extending beyond the transmembrane helices, have the potential to modulate the flexibility, spatial organization and conformational dynamics of TNFR1 in a membrane context.

### 3.4 Macroscopic motions and structural flexibility of TNR1

**Fig. 8:**
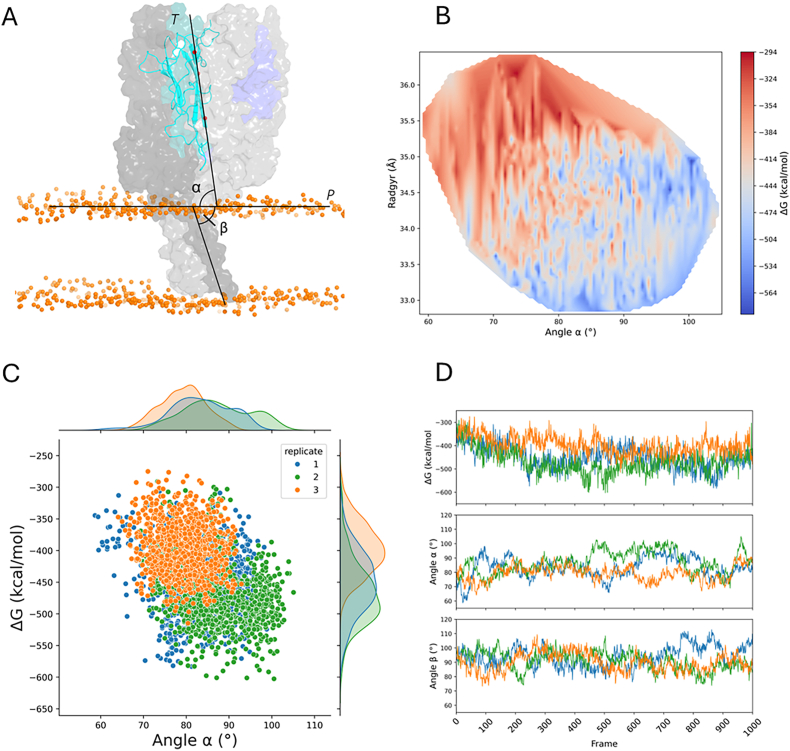
Illustration of the definition of the alpha and beta angles used to study the system's movements relative to the membrane (A). Energy landscape explored by the system as a function of the radius of gyration and the alpha angle (B). Distribution of the interaction ΔG and the alpha angle observed throughout the simulations (C). Time series of ΔG, alpha angle, and beta angle for the three replicates performed (D).

To better characterize the macroscopic motions of the system, we defined two geometric metrics to monitor the behavior of the complex. Fig. 8A illustrates the definition of the alpha and beta angles, which describe the inclination of the extracellular domains and the transmembrane helices, respectively, relative to the membrane plane.

Across all three replicates (Fig. 8B), the radius of gyration progressively decreases over the course of the trajectory, consistent with the compaction observed in the PCA analysis. More favorable ΔG values are associated with alpha angles greater than approximately 75° (Fig. 8C). The distribution of the alpha angle is highly similar in replicates 1 and 2, but differs markedly in replicate 3.

As previously noted, replicate 3 exhibited a less favorable overall ΔG, a trend that is also reflected here. Replicates 1 and 2 explore a broader range of inclinations of the extracellular region, allowing access to more energetically favorable conformations. By contrast, replicate 3 shows restricted sampling of alpha angles, which rarely exceeds 90° and seldom reaches total ΔG values below −500 kcal/mol.

For the beta angle, we observed a similar trend to the alpha angle, albeit the effect is less pronounced. Its distributions remain consistent across all three replicates, suggesting a more conserved conformation and higher rigidity in the transmembrane region.

Building on the analysis of the beta angle, we next investigated potential conformational changes within the transmembrane region to further characterize its structural behavior. To this end, we applied the HELANAL method to monitor standard helical parameters over time (Fig. S2).

Overall, the transmembrane helices exhibit stable helical characteristics throughout the simulations. However, localized and transient deviations were observed. In replicate 1, around 100 ns, we noted a transient increase in average helix height (from 1.5 Å to nearly 1.7 Å), a decrease in the number of residues per turn (from 3.60 to 3.40), and an increase in average twist angle (from 100° to 106°). These values returned to baseline by 200 ns. During this interval, corresponding fluctuations were also observed in the alpha angle, which shifted from 60° to 100° before stabilizing near 90° (Fig. 7D). In this case, the deformations were more pronounced in helix 1. A similar transient phenomenon occurred in replicate 2, where helix 2 exhibited structural alterations around 400 ns. These included a reduction in residues per turn (from 3.60 to 3.42) and an increase in twist (from 100° to 106°), accompanied by an increase in the alpha angle. In contrast, replicate 3 showed fewer such events, indicating a more structurally stable transmembrane region over the course of the simulation.

## 4 Discussion

### 4.1 Major individual contributors on TNFR1

This study enabled an in-depth characterization of the TNFR1:TNFα complex in a membrane context using enhanced all-atom molecular dynamics simulations. We examined a system composed of a trimeric ligand and a trimeric receptor embedded in a lipid bilayer, analyzing energetic contributions, key interaction sites, and the dynamic behavior influenced by the membrane environment.

Our analysis began with the decomposition of the TNFR1–TNFα interface, highlighting residues that consistently contribute to binding free energy. The all-atom GaMD approach allowed us to resolve the energetic landscape at the residue level, providing a foundation for identifying potential therapeutic targets. Notably, the majority of energetically significant residues on TNFR1 were located in CRD2 (L96, R97, E85, L100, K104, and R106), while CRD3 also featured key hydrophobic contributors (L104 and W136). In CRD4, R175 emerged as a functionally interesting residue: although not in contact with TNFα in the initial structure, it progressively established interactions with E183 through conformational reorganization. This behavior supports earlier MD simulations reporting the role of R175 (R146 in the PDB) in anchoring TNFα via CRD4 (Mascarenhas and Kästner, 2012), suggesting this domain may serve as an additional ligand-contact point beyond the canonical CRDs.

This energetic mapping intersects meaningfully with known pathogenic variants catalogued in Ensembl and ClinVar, many of which are associated with TNF Receptor-Associated Periodic Syndrome (TRAPS) (Cudrici et al., 2020), reinforcing the physiological relevance of the identified interaction hotspots. Within CRD1, variants of residue D71 (including deletions and substitutions to glycine or valine) are reported in patients and predicted to be damaging by PolyPhen-2 (Adzhubei et al., 2010) and SIFT (Vaser et al., 2016). While no known pathological mutations have been documented in CRD3, the key residue L140 is only observed in benign variants (to isoleucine or proline), remaining within the hydrophobic aliphatic class. Nevertheless, mutations in neighboring residues (H134, E138, and F141) are annotated in TRAPS cases, suggesting the broader regional importance of this domain. In CRD4, the energetically significant residue R175 appears in the variant R175G, which is also predicted to be deleterious by SIFT. The majority of high-contributing residues identified in this study, however, lie within CRD2. Among these, R106 is considered likely pathogenic in the R106Q variant. R97 is involved in a deletion mutation encompassing residues 97–100, also linked to TRAPS, along with surrounding residues. Mutation L96P has been described as deleterious and probably damaging, and linked to TRAPS. Other residues we identified as energetically important, including E85, L100, and K104, have not been directly implicated in disease variants or are considered benign (e.g., the homologous E85D variant). Nonetheless, each of these residues is in close spatial proximity to cysteines whose mutations are associated with TRAPS, such as C84 (near E85), C99 and C102 (near L100), and C105 (near K104). This enrichment of pathogenic variants and energetically important residues in cysteine-rich regions highlights the likely importance of local structural integrity and disulfide-bond-mediated rigidity in TNFR1 function. One residue mutation, R92Q, has been identified by genome-wide association study as a risk factor for multiple sclerosis (Caminero et al., 2011) but our analysis has not revealed a significant energy contribution for this residue.

The evaluation of each key residue must be considered in the context of the three main strategies for modulating the TNFR1 pathway, as outlined in previous studies (Lo, 2025): (i) direct neutralization of receptor–ligand interactions, (ii) interference with receptor–receptor interactions via the PLAD domain, or (iii) modulation of TNFR1 dynamics. Current therapeutic approaches illustrate these strategies. For example, antibodies used in certain inflammatory pathologies act on the outer surface of TNFα, thereby disrupting ligand–receptor binding, a mechanism also shared by several reported small-molecule antagonists (Chen et al., 2017; Saddala and Huang, 2019). In addition, antibodies specifically targeting TNFR1 have been developed (Richter et al., 2021), and chemical compounds have been identified that interfere with the trimerization interface of TNFα, destabilizing the ligand–receptor complex (McMillan et al., 2021). More recently, a peptide has been designed to block a specific conformational state of TNFR1, impairing signal transduction without directly disrupting TNFR1:TNFR1 or TNFR1:TNFα interactions (Zeng et al., 2024). A summary of these biologics, their binding sites, and targeted mechanisms is provided in the supplementary data (Table S3). Building on these therapeutic strategies (Zeng et al., 2024), our findings may provide complementary guidance by identifying residues that (i) influence TNFα residues critical for cytokine trimerization or TNFR1 residues essential for ligand binding, thereby modulating receptor–ligand interactions, or (ii) resides within the CRD1 domain, whose destabilization could impact receptor dimerization and consequently affect both receptor–ligand and receptor–receptor interactions.

A future direction could include large-scale in silico mutagenesis to evaluate the energetic and structural consequences of TNFR1 mutations, which may offer insights into both disease mechanisms and the therapeutic potential of targeting these domains. This calls for an enhanced effort toward systematic and recurrent evaluation of variants against continuously updated knowledge from the literature and databases, to facilitate the identification of pathogenic mutations and their associations with diverse pathologies.

### 4.2 Receptor preferences and structural constraints

Our lipid interaction analysis revealed that several phosphatidylcholine-binding clusters are located at the interface between the transmembrane and globular domains, involving charged or polar residues from the linker regions. These interactions displayed significant residence times and surface contact areas, indicating that they are not transient but rather structurally stabilizing features. This suggests a previously underappreciated role of membrane headgroup interactions in anchoring and orienting the extracellular domain, beyond the hydrophobic embedding of transmembrane helices. These linker–lipid contacts may serve as pivot points modulating receptor tilting and thereby indirectly influencing ligand engagement and signal propagation. Besides, the PCA results highlighted that linker flexibility constitutes a major component of the global motions in this system. While the globular extracellular region remains structurally stable, the linkers exhibit conformational plasticity, contributing to major principal components. This flexibility allows for repositioning of the extracellular domains relative to the membrane, which could be functionally important for receptor clustering, ligand engagement, or downstream signaling. These results complement previous observations that extracellular domain tilting modulates receptor activation states (Westerfield and Barrera, 2020), and highlight a role for the membrane environment in constraining or enabling such motions.

The three simulation replicates demonstrated marked differences in both global ΔG and the angle sampling. Replicates 1 and 2, which sampled a wider range of inclinations of the extracellular domains, reached more favorable energetic states than replicate 3, which remained more rigid. This variability underscores the importance of conformational sampling in assessing interaction energetics, and suggests that TNFR1 can adopt multiple functionally relevant orientations relative to the membrane. In a physiological context, such dynamics could be related to different activation or clustering states of the receptor under varying membrane tensions or microenvironments.

In light of previous studies on TNF receptor signaling (Su and Wu, 2020, 2023; Su et al., 2022), we considered the preference of TNFR1 for sTNFα and of TNFR2 for mbTNFα. Their multi-scale modelling studies showed that membrane anchoring of the ligand restricts receptor clustering, and suggested that TNFR2 forms larger receptor clusters under specific cis-interaction conditions, though direct structural evidence is lacking. Our comparative sequence analysis showed that most of the top TNFR1 contact residues are not conserved in TNFR2, except E85 (identical), K104 (mutated to arginine) and L96 (mutated to valine) possibly impacting ligand selectivity. Notably, TNFR1 features a shorter extracellular-to-transmembrane linker than TNFR2. Given the marked differences in global motion and energetic profiles observed across replicates, such topological constraints may influence the preferential activation of the receptor by sTNFα over mbTNFα.

Future studies aimed at modeling the TNFR1–TNFα system in its entirety, including the extracellular domain, transmembrane segments, and the full intracellular portion along with cytosolic partners, would represent a significant milestone in rationalizing TNFR1 signaling. Nowadays, achieving such a complete model remains a major challenge, as only certain regions of the system have been experimentally resolved, and the flexible part of intracellular domains are poorly characterized. Future work would require to include full-length receptor modelling, if possible with downstream signaling partners, to fully capture the consequences of ligand binding on transmembrane signal transduction, providing a more comprehensive understanding of TNFR1 activation and its pathological variants.

### 4.3 Energetic and structural determinants on the ligand side

On the TNFα side, our analysis confirmed the importance of residues within the TNF homology domain (THD), a region increasingly recognized as a druggable interface in complement to more traditional anti-TNFα therapies on the market (primarily monoclonal antibodies binding to the external surface of TNFα that is accessible to both soluble and membrane-bound forms). Notably, Y195 formed conserved П-П interactions stabilizing the trimer, consistent with previous structural reports (Eck and Sprang, 1989). Targeting this region, as proposed in recent studies using small molecules or bicyclic peptides (O'Connell et al., 2019), may enable selective disruption of sTNFα function without affecting membrane-bound TNF, offering a promising route for isoform-specific treatment.

Additionally, key residues mediating receptor interactions, such as A221, I73, L151, and Y163, were identified through energy decomposition and interaction conservation (Table S3). Several have been implicated in mutational studies that impact TNFα signaling pathways (Steed et al., 2003), reinforcing their potential as biomarkers or therapeutic targets.

## 5 Conclusion

Our simulations offer a high-resolution perspective on the TNFα–TNFR1 system within a membrane context, revealing mechanistic insights into receptor-ligand interactions and the modulatory role of the lipid environment.

We identified specific lipid–protein interaction clusters that extend beyond the transmembrane region into the flexible linker connecting to the globular domain. Principal Component Analysis (PCA) demonstrated that these linker regions exhibit the most pronounced dynamics, suggesting that membrane-associated interactions may contribute to receptor positioning and anchoring at the membrane interface.

Focusing on the TNFα–TNFR1 interface, we mapped the energetic contributions of individual residues across the Cysteine-Rich Domains (CRDs), confirming the predominant role of CRD2 while also identifying key contacts in other CRDs. Notably, several of the energetically significant residues coincide with known pathogenic mutation sites, highlighting their functional and clinical relevance.

Finally, by examining intra-trimeric interactions within TNFα, we characterized a conserved aromatic and hydrophobic core essential for trimer stability. This detailed characterization not only enhances our understanding of TNFα structure but also provides a framework for the rational design of molecules that modulate TNF signaling through oligomeric stabilization or disruption.

Altogether, this work offers a multidimensional view of the TNFα–TNFR1 complex, integrating membrane effects, conformational dynamics, and molecular energetics, and sets the stage for future structure-guided studies aimed at therapeutic modalities.

## Credit author statement

ST and SH did the conceptualization and funding acquisition of the study; EAS and ST set up the Methology of the study; EAS did the Data curation, the formal analysis of the results, and their visualization; EAS, SH and ST did the writing – original draft and writing – review and editing. All authors have read and accepted the manuscript in the present form.

## Declaration of competing interest

The authors declare the following financial interests/personal relationships which may be considered as potential competing interests:Elena Alvarez Sanchez reports financial support was provided by Affilogic SAS. Stephane TELETCHEA reports a relationship with Nantes University that includes: funding grants. Elena Alvarez Sanchez PhD salary is supported by the Affilogic SAS company. If there are other authors, they declare that they have no known competing financial interests or personal relationships that could have appeared to influence the work reported in this paper.

## Figures and Tables

**figs1:**
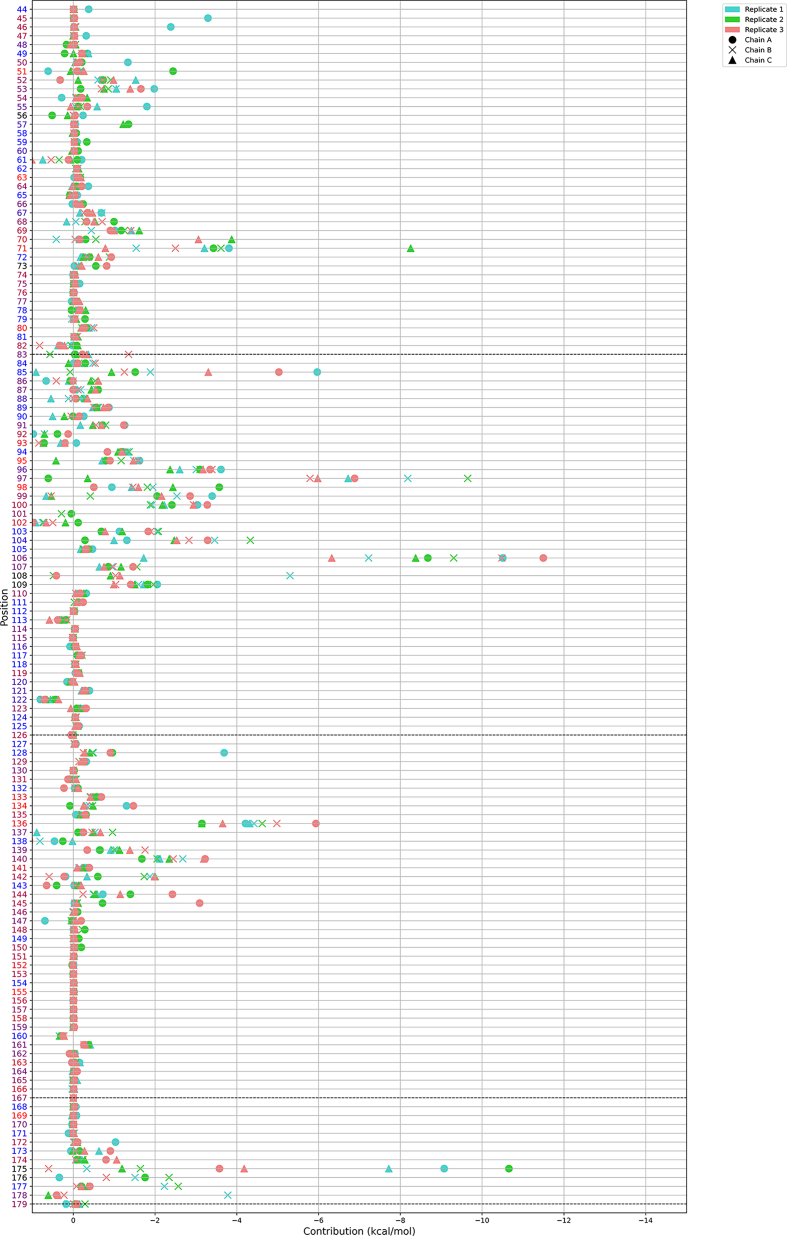


**figs2:**
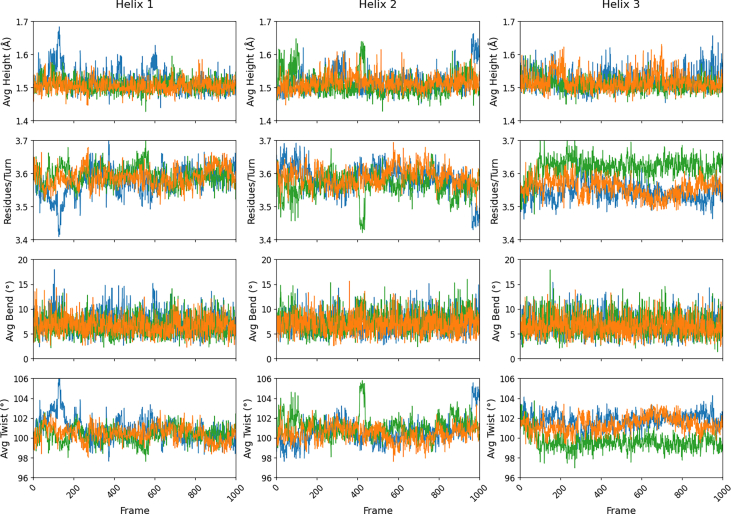


**figs3:**
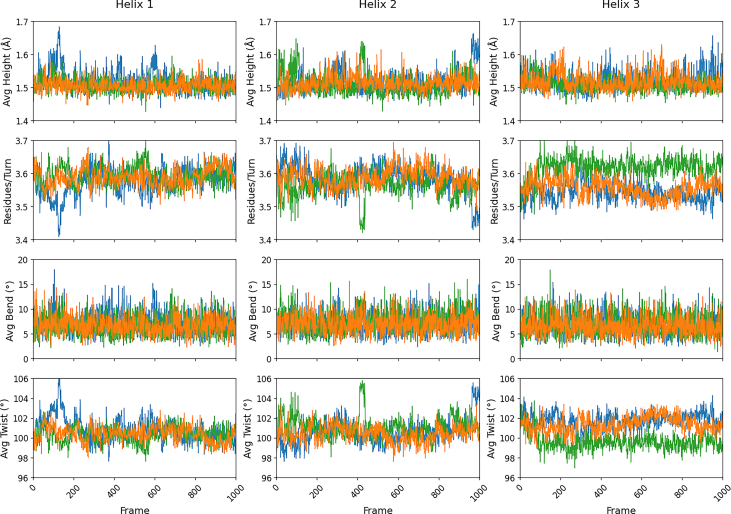


## References

[bib1] Adzhubei I.A., Schmidt S., Peshkin L., Ramensky V.E., Gerasimova A., Bork P., Kondrashov A.S., Sunyaev S.R. (2010). A method and server for predicting damaging missense mutations. Nat. Methods..

[bib2] Aebisher D., Bartusik-Aebisher D., Przygórzewska A., Oleś P., Woźnicki P., Kawczyk-Krupka A. (2024). Key interleukins in inflammatory bowel Disease-A review of recent studies. Int. J. Mol. Sci..

[bib3] Banner D.W., D'Arcy A., Janes W., Gentz R., Schoenfeld H.J., Broger C., Loetscher H., Lesslauer W. (1993). Crystal structure of the soluble human 55 kd TNF receptor-human TNF beta complex: implications for TNF receptor activation. Cell..

[bib4] Bansal M., Kumar S., Velavan R. (2000). HELANAL: a program to characterize helix geometry in proteins. J. Biomol. Struct. Dyn..

[bib5] Caminero A., Comabella M., Montalban X. (2011). Role of tumour necrosis factor (TNF)-α and TNFRSF1A R92Q mutation in the pathogenesis of TNF receptor-associated periodic syndrome and multiple sclerosis. Clin. Exp. Immunol..

[bib6] Carswell E.A., Old L.J., Kassel R.L., Green S., Fiore N., Williamson B. (1975). An endotoxin-induced serum factor that causes necrosis of tumors. Proc. Natl. Acad. Sci. U. S. A..

[bib7] Case D.A., Cheatham T.E., Darden T., Gohlke H., Luo R., Merz K.M., Onufriev A., Simmerling C., Wang B., Woods R.J. (2005). The Amber biomolecular simulation programs. J. Comput. Chem..

[bib8] Case D.A., Aktulga H.M., Belfon K., Cerutti D.S., Cisneros G.A., Cruzeiro V.W.D., Forouzesh N., Giese T.J., Götz A.W., Gohlke H., Izadi S., Kasavajhala K., Kaymak M.C., King E., Kurtzman T., Lee T.-S., Li P., Liu J., Luchko T., Luo R., Manathunga M., Machado M.R., Nguyen H.M., O'Hearn K.A., Onufriev A.V., Pan F., Pantano S., Qi R., Rahnamoun A., Risheh A., Schott-Verdugo S., Shajan A., Swails J., Wang J., Wei H., Wu X., Wu Y., Zhang S., Zhao S., Zhu Q., Cheatham T.E., Roe D.R., Roitberg A., Simmerling C., York D.M., Nagan M.C., Merz K.M. (2023). AmberTools. J. Chem. Inf. Model..

[bib9] Chen S., Feng Z., Wang Y., Ma S., Hu Z., Yang P., Chai Y., Xie X. (2017). Discovery of novel ligands for TNF-α and TNF Receptor-1 through structure-based virtual screening and biological assay. J. Chem. Inf. Model..

[bib10] Cudrici C., Deuitch N., Aksentijevich I. (2020). Revisiting TNF receptor-associated periodic syndrome (TRAPS): current perspectives. Int. J. Mol. Sci..

[bib11] Del Conte A., Camagni G.F., Clementel D., Minervini G., Monzon A.M., Ferrari C., Piovesan D., Tosatto S.C.E. (2024). Ring 4.0: faster residue interaction networks with novel interaction types across over 35,000 different chemical structures. Nucleic Acids Res..

[bib12] Dickson C.J., Walker R.C., Gould I.R. (2022). Lipid21: complex lipid membrane simulations with AMBER. J. Chem. Theor. Comput..

[bib13] Ding Q., Hu W., Wang R., Yang Q., Zhu M., Li M., Cai J., Rose P., Mao J., Zhu Y.Z. (2023). Signaling pathways in rheumatoid arthritis: implications for targeted therapy. Signal Transduct. Targeted Ther..

[bib14] Eck M.J., Sprang S.R. (1989). The structure of tumor necrosis factor-alpha at 2.6 A resolution. Implications for receptor binding. J. Biol. Chem..

[bib15] Fiser A., Sali A. (2003). ModLoop: automated modeling of loops in protein structures. Bioinformatics..

[bib16] Gowers R.J., Linke M., Barnoud J., Reddy T.J.E., Melo M.N., Seyler S.L., Domański J., Dotson D.L., Buchoux S., Kenney I.M., Beckstein O. (2016). MDAnalysis: a python package for the rapid analysis of molecular dynamics simulations. Scipy..

[bib17] Grant B.J., Skjaerven L., Yao X.-Q. (2021). The Bio3D packages for structural bioinformatics. Protein Sci..

[bib18] Hu S., Liang S., Guo H., Zhang D., Li H., Wang X., Yang W., Qian W., Hou S., Wang H., Guo Y., Lou Z. (2013). Comparison of the inhibition mechanisms of adalimumab and infliximab in treating tumor necrosis factor α-associated diseases from a molecular view. J. Biol. Chem..

[bib19] Huang P.-S., Ban Y.-E.A., Richter F., Andre I., Vernon R., Schief W.R., Baker D. (2011). RosettaRemodel: a generalized framework for flexible backbone protein design. PLoS One..

[bib20] Jang D., Lee A.-H., Shin H.-Y., Song H.-R., Park J.-H., Kang T.-B., Lee S.-R., Yang S.-H. (2021). The role of tumor necrosis factor alpha (TNF-α) in autoimmune disease and current TNF-α inhibitors in therapeutics. Int. J. Mol. Sci..

[bib21] Kaufmann K.W., Lemmon G.H., Deluca S.L., Sheehan J.H., Meiler J. (2010). Practically useful: what the rosetta protein modeling suite can do for you. Biochem..

[bib22] Lejeune F.J., Liénard D., Matter M., Rüegg C. (2006). Efficiency of recombinant human TNF in human cancer therapy. Cancer Immun..

[bib23] Lewis A.K., Valley C.C., Sachs J.N. (2012). TNFR1 signaling is associated with backbone conformational changes of receptor dimers consistent with overactivation in the R92Q TRAPS mutant. Biochem..

[bib24] Liang S., Dai J., Hou S., Su L., Zhang D., Guo H., Hu S., Wang H., Rao Z., Guo Y., Lou Z. (2013). Structural basis for treating tumor necrosis factor α (TNFα)-associated diseases with the therapeutic antibody infliximab. J. Biol. Chem..

[bib25] Lo C.H. (2025). TNF receptors: structure-function relationships and therapeutic targeting strategies. Biochim. Biophys. Acta Biomembr..

[bib26] Lomize M.A., Pogozheva I.D., Joo H., Mosberg H.I., Lomize A.L. (2012). OPM database and PPM web server: resources for positioning of proteins in membranes. Nucleic Acids Res..

[bib27] Maier J.A., Martinez C., Kasavajhala K., Wickstrom L., Hauser K.E., Simmerling C. (2015). ff14SB: improving the accuracy of protein side chain and backbone parameters from ff99SB. J. Chem. Theor. Comput..

[bib28] Mark P., Nilsson L. (2002). Structure and dynamics of liquid water with different long-range interaction truncation and temperature control methods in molecular dynamics simulations. J. Comput. Chem..

[bib29] Mascarenhas N.M., Kästner J. (2012). Are different stoichiometries feasible for complexes between lymphotoxin-alpha and tumor necrosis factor receptor 1?. BMC Struct. Biol..

[bib30] McDermott M.F., Aksentijevich I., Galon J., McDermott E.M., Ogunkolade B.W., Centola M., Mansfield E., Gadina M., Karenko L., Pettersson T., McCarthy J., Frucht D.M., Aringer M., Torosyan Y., Teppo A.M., Wilson M., Karaarslan H.M., Wan Y., Todd I., Wood G., Schlimgen R., Kumarajeewa T.R., Cooper S.M., Vella J.P., Amos C.I., Mulley J., Quane K.A., Molloy M.G., Ranki A., Powell R.J., Hitman G.A., O'Shea J.J., Kastner D.L. (1999). Germline mutations in the extracellular domains of the 55 kDa TNF receptor, TNFR1, define a family of dominantly inherited autoinflammatory syndromes. Cell.

[bib31] McMillan D., Martinez-Fleites C., Porter J., Fox D., Davis R., Mori P., Ceska T., Carrington B., Lawson A., Bourne T., O'Connell J. (2021). Structural insights into the disruption of TNF-TNFR1 signalling by small molecules stabilising a distorted TNF. Nat. Commun..

[bib32] Miao Y., McCammon J.A. (2017). Gaussian accelerated molecular dynamics: theory, implementation, and applications. Annu. Rep. Comput. Chem..

[bib33] Michaud-Agrawal N., Denning E.J., Woolf T.B., Beckstein O. (2011). MDAnalysis: a toolkit for the analysis of molecular dynamics simulations. J. Comput. Chem..

[bib34] Miller B.R., McGee T.D., Swails J.M., Homeyer N., Gohlke H., Roitberg A.E. (2012). MMPBSA.py: an efficient program for end-state free energy calculations. J. Chem. Theor. Comput..

[bib35] Naismith J.H., Devine T.Q., Brandhuber B.J., Sprang S.R. (1995). Crystallographic evidence for dimerization of unliganded tumor necrosis factor receptor. J. Biol. Chem..

[bib36] Ono M., Horita S., Sato Y., Nomura Y., Iwata S., Nomura N. (2018). Structural basis for tumor necrosis factor blockade with the therapeutic antibody golimumab. Protein Sci..

[bib37] O'Connell J., Porter J., Kroeplien B., Norman T., Rapecki S., Davis R., McMillan D., Arakaki T., Burgin A., Fox D., Ceska T., Lecomte F., Maloney A., Vugler A., Carrington B., Cossins B.P., Bourne T., Lawson A. (2019). Small molecules that inhibit TNF signalling by stabilising an asymmetric form of the trimer. Nat. Commun..

[bib38] Palladino M.A., Bahjat F.R., Theodorakis E.A., Moldawer L.L. (2003). Anti-TNF-α therapies: the next generation. Nat. Rev. Drug Discov..

[bib39] Richardson J.S. (1981). The anatomy and taxonomy of protein structure. Adv. Protein Chem..

[bib40] Richter F., Williams S.K., John K., Huber C., Vaslin C., Zanker H., Fairless R., Pichi K., Marhenke S., Vogel A., Dhaen M.-A., Herrmann S., Herrmann A., Pfizenmaier K., Bantel H., Diem R., Kontermann R.E., Fischer R. (2021). The TNFR1 antagonist atrosimab is therapeutic in mouse models of acute and chronic inflammation. Front. Immunol..

[bib41] Roe D.R., Cheatham T.E. (2013). PTRAJ and CPPTRAJ: software for processing and analysis of molecular dynamics trajectory data. J. Chem. Theor. Comput..

[bib42] Ruder B., Atreya R., Becker C. (2019). Tumour necrosis factor alpha in intestinal homeostasis and gut related diseases. Int. J. Mol. Sci..

[bib43] Saddala M.S., Huang H. (2019). Identification of novel inhibitors for TNFα, TNFR1 and TNFα-TNFR1 complex using pharmacophore-based approaches. J. Transl. Med..

[bib44] Sagui C., Pedersen L.G., Darden T.A. (2004). Towards an accurate representation of electrostatics in classical force fields: efficient implementation of multipolar interactions in biomolecular simulations. J. Chem. Phys..

[bib45] Sandborn W.J., Hanauer S.B., Katz S., Safdi M., Wolf D.G., Baerg R.D., Tremaine W.J., Johnson T., Diehl N.N., Zinsmeister A.R. (2001). Etanercept for active Crohn's disease: a randomized, double-blind, placebo-controlled trial. Gastroenterology.

[bib46] Schott-Verdugo S., Gohlke H. (2019). PACKMOL-Memgen: a simple-to-use, generalized workflow for membrane-protein–lipid-bilayer system building. J. Chem. Inf. Model..

[bib47] Smith R.A., Baglioni C. (1987). The active form of tumor necrosis factor is a trimer. J. Biol. Chem..

[bib48] Song W., Corey R.A., Ansell T.B., Cassidy C.K., Horrell M.R., Duncan A.L., Stansfeld P.J., Sansom M.S.P. (2022). PyLipID: a python package for analysis of protein-lipid interactions from molecular dynamics simulations. J. Chem. Theor. Comput..

[bib49] Steed P.M., Tansey M.G., Zalevsky J., Zhukovsky E.A., Desjarlais J.R., Szymkowski D.E., Abbott C., Carmichael D., Chan C., Cherry L., Cheung P., Chirino A.J., Chung H.H., Doberstein S.K., Eivazi A., Filikov A.V., Gao S.X., Hubert R.S., Hwang M., Hyun L., Kashi S., Kim A., Kim E., Kung J., Martinez S.P., Muchhal U.S., Nguyen D.-H.T., O'Brien C., O'Keefe D., Singer K., Vafa O., Vielmetter J., Yoder S.C., Dahiyat B.I. (2003). Inactivation of TNF signaling by rationally designed dominant-negative TNF variants. Science.

[bib50] Su Z., Wu Y. (2020). Computational simulations of TNF receptor oligomerization on plasma membrane. Proteins: Struct., Funct., Bioinf..

[bib51] Su Z., Wu Y. (2023). How does the same ligand activate signaling of different receptors in TNFR superfamily: a computational study. J. Cell Commun. Signal..

[bib52] Su Z., Dhusia K., Wu Y. (2022). Understanding the functional role of membrane confinements in TNF-mediated signaling by multiscale simulations. Commun. Biol..

[bib53] van Loo G., Bertrand M.J.M. (2023). Death by TNF: a road to inflammation. Nat. Rev. Immunol..

[bib54] Vaser R., Adusumalli S., Leng S.N., Sikic M., Ng P.C. (2016). SIFT missense predictions for genomes. Nat. Protoc..

[bib55] Westerfield J.M., Barrera F.N. (2020). Membrane receptor activation mechanisms and transmembrane peptide tools to elucidate them. J. Biol. Chem..

[bib56] Yang S., Wang J., Brand D.D., Zheng S.G. (2018). Role of TNF-TNF receptor 2 signal in regulatory T cells and its therapeutic implications. Front. Immunol..

[bib57] Zeng J., Loi G.W.Z., Saipuljumri E.N., Romero Durán M.A., Silva-García O., Perez-Aguilar J.M., Baizabal-Aguirre V.M., Lo C.H. (2024). Peptide-based allosteric inhibitor targets TNFR1 conformationally active region and disables receptor–ligand signaling complex. Proc. Natl. Acad. Sci..

