# Supplementary material for: Molecular determinants of TNFR1:TNFα binding and dynamics in a physiological membrane environment
Source: Curr Res Struct Biol. 2025 Dec 18;11:100177. doi: 10.1016/j.crstbi.2025.100177 (PMC12811475; doi:10.1016/j.crstbi.2025.100177)
Supplement: Multimedia component 1 [file mmc1.docx]

# Supplementary Figure captions

**Figure S1.** Sequence of the TNFR1 with each position colored based on sequence similarity and structural superposition relative to TNFR2. Each replicate is represented by a different color, and each chain is indicated by a different symbol. Each CRD is enclosed between two dashed lines.

**Figure S2.** Results obtained from the PCA performed on 1000 ns the first replicate. Representation of the main motions explained by PC1 (A) and PC2 (B). Vectors representing the main motion of the system (red arrows) (C). Clusters centers for the two clusters identified after PCA analysis. TNFR1 structures are colored in black for cluster 1 and in green for cluster2 (D).

**Figure S3.** Timeseries of HELANAL parameters for the three helices forming the transmembrane segments of TNFR1 for replicates 1, 2 and 3. These parameters are the following: Average Helix Height, the number of residues per turn, the average bend and the average twist.

**Table S1.** TNFR1 residues forming the protein-lipid interaction clusters 0, 1, 2, 3, 4, 5, 6, and 7 identified with PyLipID.

| **Cluster** | **Residues** |
| --- | --- |
| 0 | C168, T167, N151, C149, L150, A170, G152, H155, T153, G171, H169, V154, F172 |
| 1 | H128, I158, V555, T164, G163, K162, V161, N160, E159, Q157, A129, P156, L155, L148, S142, S140, F131, G130, L556 |
| 2 | L227, I230, L227, F229, I230, F229, L232, L228, L227, G231, L232, L228, G231, I230, L224, G231, F229, L232, S226, L225, S226, L225 |
| 3 | K193, K203, G152, E190, G204, K187, L189, L150, N201, S188, N151, L148, T153, E200, V154, C149, C191, C168, V202, C166, 192, T167, C185, Q198, C182, I199 |
| 4 | D207, G209, T210, V212, T205, E206, D207, S208, L213, S181, T210, T211, N184, S183, E206, L214, K186, K186, T211, L216, V217, F219, C182, K187, L222 |
| 5 | L214, L213, H169, G209, S208, L216, L213, P215, V212, T211, E206, D207, T205, V202, A170, T210, V217, G171, L214, G204, K203, E200, N201 |
| 6 | N151, F172, G152, T153, C168, L150, T167, I199, L148, C149, S147, C179, V154, Q198 |
| 7 | E183, C185, V180, G184, A185 |

**Table S2.** Details of the most conserved interactions between TNFR1 and TNFα. For each selected residue on TNFR1, the corresponding interacting residue on TNFα is identified. Additionally, to assess the distribution of interactions across the different protomers of TNFα, we have specified whether the interaction occurs with protomer 1 or protomer 2.

| **Position TNFR1** | **Position TNFa** | **TNFa protomer (1/2)** | **Interaction type** |
| --- | --- | --- | --- |
| D71 | R82 | 1 | HB |
| R97 | E203 | 2 | HB |
| L96 | S162 | 2 | HB |
| L96 | Y163 | 2 | VDW |
| E85 | R107 | 1 | HB |
| L100 | Y163 | 2 | VDW |
| L100 | V167 | 2 | VDW |
| K104 | A221 | 1 | HB |
| K104 | P96 | 1 | VDW |
| R106 | D219 | 1 | HB |
| L140 | I173 | 2 | VDW |
| W136 | L151 | 2 | VDW |
| W136 | N213 | 2 | HB |
| R175 | E183 | 1 | Ionic+HB+VDW |
