# Supplementary material for: Molecular determinants of TNFR1:TNFα binding and dynamics in a physiological membrane environment
Source: Curr Res Struct Biol. 2025 Dec 18;11:100177. doi: 10.1016/j.crstbi.2025.100177 (PMC12811475; doi:10.1016/j.crstbi.2025.100177)
Supplement: Multimedia component 2 [file mmc2.docx]

**Table S1.** TNFR1 residues forming the protein-lipid interaction clusters 0, 1, 2, 3, 4, 5, 6, and 7 identified with PyLipID.

| **Cluster** | **Residues** |
| --- | --- |
| 0 | C168, T167, N151, C149, L150, A170, G152, H155, T153, G171, H169, V154, F172 |
| 1 | H128, I158, V555, T164, G163, K162, V161, N160, E159, Q157, A129, P156, L155, L148, S142, S140, F131, G130, L556 |
| 2 | L227, I230, L227, F229, I230, F229, L232, L228, L227, G231, L232, L228, G231, I230, L224, G231, F229, L232, S226, L225, S226, L225 |
| 3 | K193, K203, G152, E190, G204, K187, L189, L150, N201, S188, N151, L148, T153, E200, V154, C149, C191, C168, V202, C166, 192, T167, C185, Q198, C182, I199 |
| 4 | D207, G209, T210, V212, T205, E206, D207, S208, L213, S181, T210, T211, N184, S183, E206, L214, K186, K186, T211, L216, V217, F219, C182, K187, L222 |
| 5 | L214, L213, H169, G209, S208, L216, L213, P215, V212, T211, E206, D207, T205, V202, A170, T210, V217, G171, L214, G204, K203, E200, N201 |
| 6 | N151, F172, G152, T153, C168, L150, T167, I199, L148, C149, S147, C179, V154, Q198 |
| 7 | E183, C185, V180, G184, A185 |

**Table S2.** Details of the most conserved interactions between TNFR1 and TNFα. For each selected residue on TNFR1, the corresponding interacting residue on TNFα is identified. Additionally, to assess the distribution of interactions across the different protomers of TNFα, we have specified whether the interaction occurs with protomer 1 or protomer 2.

| **Position TNFR1** | **Position** TNFα | **TNFa protomer (1/2)** | **Interaction type** |
| --- | --- | --- | --- |
| D71 | R82 | 1 | HB |
| R97 | E203 | 2 | HB |
| L96 | S162 | 2 | HB |
| L96 | Y163 | 2 | VDW |
| E85 | R107 | 1 | HB |
| L100 | Y163 | 2 | VDW |
| L100 | V167 | 2 | VDW |
| K104 | A221 | 1 | HB |
| K104 | P96 | 1 | VDW |
| R106 | D219 | 1 | HB |
| L140 | I173 | 2 | VDW |
| W136 | L151 | 2 | VDW |
| W136 | N213 | 2 | HB |
| R175 | E183 | 1 | Ionic+HB+VDW |

**Table S3.** Details of different molecules targeting TNFα or TNFR1 in order to affect their interaction. The directly targeted system is specified. The binding site is also indicated using the Uniprot numbering for molecules with an experimentally solved or predicted structure (T1, R1, Astrosab, or FKC peptide).

| Molecule | Targeted interaction | Targeted molecule | Binding site | PDB code |
| --- | --- | --- | --- | --- |
| SPD-304 | TNFα- TNFα | TNFα | L133 Y135 Y195 G197 Y227 | 2AZ5 |
| T1 | TNFα- TNFα | TNFα | L133 Y135 Y195 G197 Y227 | predicted |
| R1 | TNFα-TNFR1 | TNFR1 | A91 H95 | predicted |
| Astrosab | TNFR1-TNFα | TNFR1 | P52 L96 R97 H98 | predicted |
| FKC peptide | TNFR1-TNFR1 | TNFR1 | D122, Y135, S137, F144, Q159, K161, N163, T164, E176, N177 | predicted |
| UCB-0595 | TNFα- TNFα | TNFα | G197 Y227 Y135 L223 Y195 | 7KP8 |
| Adalimumab | TNFα-TNFR1 | TNFα | Q143 P146 S147 H149 T181 E183 N213 R214 D216 Y217 | 3WD5 |
| Infliximab | TNFα-TNFR1 | TNFα | D216 N213 T153 H149 P146 R214 D216 K188 | 4G3Y |
| Certolizumab | TNFα-TNFR1 | TNFa | R207 Q164 S162 R120 Q123 N213 R214 | 5WUX |
| Golimumab | TNFα-TNFR1 | TNFa | G184 T181 E180 Q143 K141 Q143 S147 R214 Y214 | 5YOY |
